# Challenges faced by staff managing learners with spina bifida in South African special schools

**DOI:** 10.4102/hsag.v30i0.2993

**Published:** 2025-09-09

**Authors:** Sasavona R. Mashamba, Saajida Mahomed, Jacqueline M. van Wyk

**Affiliations:** 1 School of Medicine, College of Health Sciences, University of KwaZulu-Natal, Durban, South Africa; 2 Department of Education, Tshilidzini Special School, Thohoyandou, South Africa; 3 Department of Health Science Education, Faculty of Health Sciences, University of Cape Town, Cape Town, South Africa

**Keywords:** inclusive education, medical needs, training, resource allocation, infection control, educators, academic performance

## Abstract

**Background:**

Special schools in South Africa play a dual role in education and healthcare for learners with disabilities, including those with spina bifida. However, the challenges faced by school staff in managing these learners are not well documented.

**Aim:**

This study explored the challenges faced by staff in managing learners with spina bifida in special school settings and gathered their recommendations for improving care and management.

**Setting:**

Special schools across two South African provinces.

**Methods:**

A qualitative descriptive case study approach was employed. Data were collected using an open-ended interview guide with 41 purposively selected participants: 22 educators, 7 health staff, 7 allied health professionals, and 5 housemothers. Follow-up in-depth interviews were conducted with 15 of the 41 participants: 6 educators, 3 allied health professionals, 4 health staff and 2 housemothers to further explore key concerns and reconsiderations. Thematic analysis was used to analyse data.

**Results:**

Staff reported significant challenges, including insufficient resources, a lack of clear health protocols, and poor academic outcomes for learners with spina bifida. Limited self-care skills and independence among learners, often exacerbated by parental neglect, were also highlighted. Participants emphasised the need for developmental support and stronger multidisciplinary collaboration.

**Conclusion:**

Addressing the complex needs of learners with spina bifida in special schools required targeted resource allocation, robust health protocols, and integrated support systems.

**Contribution:**

The study offers practical insights into improving the management and support of learners with spina bifida in special schools, contributing to policy and practice in low-and middle-income settings like South Africa.

## Introduction

### Background

Spina bifida is a congenital condition that results in various physical, cognitive and medical challenges (Liptak & El Samra 2010). Despite advances in antenatal care and surgical interventions, which have significantly improved survival rates (Karsonovich, Brea & Munakomi 2025), learners with spina bifida (LSB) continue to face complex barriers to full participation in educational settings.

Globally, spina bifida affects approximately 1 in every 1000 births (Copp et al. 2015). Prevalence rates are higher in developing regions such as sub-Saharan Africa and Southeast Asia, ranging from 1.5 to 3 per 1000 births. In South Africa, the estimated prevalence is lower, at 0.77 per 1000 (Fieggen & Stewrt 2014), a reduction likely linked to the country’s folic acid fortification programme (Sayed et al. 2008). However, rural areas continue to report higher incidence rates (Robertson 1997), and managing the needs of LSB remains a significant public health and educational challenge (Banda 2016).

In South Africa, most LSB attend special schools, which serve as both educational institutions and providers of essential healthcare and rehabilitation services, including physiotherapy, speech therapy and audiology (Department of Basic Education 2001; Hodgson 2017). These schools operate within the inclusive education framework articulated in Education White Paper 6 (Department of Basic Education 2001), which promotes the integration of learners with special needs into mainstream settings, supported by full-service schools. Nevertheless, the severity and complexity of conditions such as spina bifida often necessitate specialised placements (World Health Organization 2011).

Managing the needs of LSB in these contexts involves a multidisciplinary team. Educators are responsible for academic development, nurses monitor physical health and administer medical care, and allied health professionals, such as speech therapists, support mobility, functional skills and communication. Social workers address emotional well-being, while boarding staff, such as house mothers, manage day-to-day care. This holistic support aims to foster inclusion, well-being and optimal functioning (Department of Basic Education 2001; Lindsay, McPherson & Maxwell 2017).

Many LSB reside in school hostels because of long travel distances. In urban or better-resourced areas, state or non-governmental organisation sponsored transport services are available but often are unreliable and inadequately equipped, lacking trained personnel or accessible vehicles (Hodgson 2017). These structural challenges are exacerbated in under-resourced schools, particularly in rural communities with limited access to specialised healthcare and infrastructure (Donohue & Bornma 2014).

The complexity of spina bifida necessitates continuous monitoring. For example, reduced sensation in the lower limbs can lead to complications such as pressure sores, infections and deformities (Governey, Culligan & Leonard 2014; Mayo Clinic 2023). The lack of sensory feedback increases the risk of pressure injuries because of an inability to reposition the body (Apkon et al. 2014). In addition, cognitive challenges, particularly among those with hydrocephalus, can impair attention, memory, executive function and processing speed, affecting academic performance (Dennis et al. 2006).

Although inclusive education is a human right that prioritises dignity, autonomy- and well-being (United Nations 2016), specialised support is sometimes more appropriate for learners with complex disabilities. This study adopts a flexible view of inclusion, consistent with the Salamanca Statement (UNESCO 1994) and White Paper 6, recognising the need for educational environments that are responsive to diverse learner needs.

Special schools are expected to play a key role in removing barriers to learning, as outlined in the National Policy on Screening, Identification, Assessment, and Support (Department of Basic Education 2014). However, their capacity is often limited by shortages of skilled staff, infrastructure deficits and inadequate assistive technologies (Human Rights Commission 2018). These limitations constrain the delivery of comprehensive care and hinder broader inclusion efforts.

The management of LSB in South African schools remains under-researched. While policy frameworks call for inclusive practices and professional development for educators and allied staff, implementation is inconsistent and poorly monitored (Hodgson 2017). Existing studies highlight the need for greater institutional support, yet there is limited evidence on the day-to-day challenges faced by school staff in managing LSB or the effectiveness of current interventions (Mashamba, Mahomed & Van Wyk 2024). This study contributes to filling that gap. Guided by the International Classification of Functioning, Disability and Health (ICF) framework, it explores how physical, cognitive and environmental factors shape the educational experiences of LSB. In doing so, it supports sustainable development goal (SDG) 4, which advocates for inclusive, equitable and quality education for all.

### Conceptual framework

The ICF as developed by the WHO (2001) serves as a framework to understand and classify health and disability (WHO 2001). The WHO defines disability as ‘impairments, activity limitations and participation restrictions’, including ‘interactions between individuals with a health condition and personal and environmental factors. The ICF’s core domains, body functions and structures, activities, participation, environmental factors and personal factors offer a comprehensive lens to understand the challenges faced by staff managing LSB. The ICF consists of four principles that are important for its application, including universality, parity and aetiological neutrality, neutrality and environmental influence. The study’s focus on staff experiences contributes to achieving SDG 4.5 by identifying practical strategies to reduce educational disparities. By emphasising the interaction between individual and contextual factors, this research supports the global push for inclusive education and the ‘leave no one behind’ agenda, advancing disability rights and meaningful participation in educational settings.

### Aim and objectives

This study explored the challenges of staff in managing and educating LSB in South African special schools. The objectives were to:

1. To identify the barriers encountered in providing care and education to LSB.
2. To examine the specific needs of LSB in the special school settings.
3. To recommend strategies to improve the management, care, and educational outcomes of LSB.

## Methodology and study design

### Study design

This exploratory, qualitative descriptive case study was conducted in two provinces in South Africa. The qualitative design was selected as the appropriate method to answer questions relating to the experiences, meanings and perspectives of the participants who teach and provide care in special school settings (Hammarberg, Kirkman & De Lacey 2016).

### Study setting

The study was conducted in seven special schools catering to LSB across two provinces in South Africa: Limpopo and Gauteng. Limpopo is predominantly rural, with over 80.0% of its population living in rural areas (Malatji 2020). In contrast, Gauteng is an urban province where 99.6% of the population resides in cities (Levy 2014). Gauteng also includes a network of urban centres such as Johannesburg, Tshwane and Ekurhuleni (Ebrahim & Everatt 2023). Among the selected schools, three were located in Limpopo and four in Gauteng. This mix of school locations helped to capture a variety of social and environmental contexts, which adds depth to the study and makes the findings more relevant to different types of communities in the South African setting.

### Inclusion and exclusion criteria

The study included staff from schools accommodating LSB who had participated in the earlier study on knowledge, attitudes and practices regarding infection control and who provided consent to participate. Staff were excluded if they were from schools not associated with LSB or if they chose not to participate, even if their school enrolled such learners.

### Recruitment process

Participants were purposively selected from the initial study on knowledge, attitudes and practices regarding infection control for LSB. Staff members were contacted telephonically and invited to participate. Only those who provided informed consent were included in the study.

### Participants and sampling

The population of this study were purposefully selected for their experiences as educators, nurses, health promoters, physiotherapists, psychologists, speech therapists, occupational therapists, social workers, class assistants and/or house mothers, who were employed at one of the seven special schools. The word staff is used to collectively refer to these groups. In this study, a house mother refers to a woman who takes care of LSB while they reside in the school’s boarding facilities during the school term.

The sample included 41 staff members responsible for education, healthcare, rehabilitation, psychosocial support and therapeutic management. These participants were chosen for their direct involvement in the care of LSB and their ability to provide insights into the challenges staff face. The ages of participant range from 30 to 62 years and their work experience ranged from 2 to 32 years. In addition, 15 participants were selected from the initial group of 41 participants who took part in the first phase of the interviews, to participate in in-depth follow-up interviews. The ages of these participants ranged from 30 to 56 years, and their work experience ranged from 2 to 32 years. The biographical details of the participants are summarised in Table 1.

**TABLE 1:** Demographic profiles of participants of phase one of the study and in-depth interviews.

School	Participant ID	Gender	Age (years)	Occupation	Years of experience
School A	01	Female	45	Auxiliary nurse	5
	02	Female	35	Auxiliary nurse	3
	03*	Female	30	Physiotherapist	7
	04	Female	58	Housemother	15
	05*	Female	46	Educator	12
	06*	Female	56	Professional nurse	2
	07	Female	49	Housemother	7
	08*	Female	42	Housemother	14
	09	Female	56	Educator	26
	10	Male	46	Educator	16
	11	Female	50	Educator	20
	12	Male	52	Educator	13
	13	Female	49	Educator	10
School B	14	Female	44	Auxiliary nurse	7
	15*	Female	35	Social worker	6
	16	Female	42	Educator	14
	17	Female	54	Educator	22
	18*	Female	47	Auxiliary nurse	10
	19	Female	45	Housemother	13
School C	20*	Female	55	Housemother	25
	21	Female	62	Educator	32
	22*	Female	35	Professional nurse	5
	23*	Female	45	Educator	11
School D	24*	Female	54	Educator	22
	25*	Female	55	Educator	27
School E	26*	Female	30	Educator	3
	27	Female	35	Physiotherapist	10
	28	Female	51	Educator	5
	29	Female	45	Educator	7
	30*	Female	56	Professional nurse	32
	31	Male	40	Educator	6
	32*	Female	43	Occupational therapist	10
	33	Female	49	Speech therapist	16
	34	Male	43	Educator	10
	35	Female	48	Educator	7
School F	36	Male	49	Educator	18
	37*	Female	55	Educator	21
	38	Female	49	Educator	9
School G	39	Female	48	Occupational therapist	16
	40	Female	53	Occupational therapist	23
	41	Female	49	Educator	11

Note:

* , Participants who took part in in-depth interviews are marked with an asterisk.

### Data collection

The interview guide was developed in English by the principal researcher with input from the supervisors. It was informed by a review of relevant literature and previous research on the study topic. The interview guide was pretested with five individuals working in the school accommodating LSB. The school and participants were not included in the main study. Feedback from the pilot test was used to revise and improve the questions. The wordings of some questions were revised to make them clearer and easier for participants to understand. Data collection occurred from July 2021 to September 2022 during the coronavirus disease 2019 (COVID-19) lockdown. Information about the study was communicated to participants by telephone. Participant information had been collected during the initial phase of study that explored knowledge, attitudes and practices regarding infection control for LSB and only those who consented were invited for follow-up in-depth interview. All procedures were conducted compliance with the requirements of the *Protection of Personal Information Act* (POPIA). Data were stored securely, and participants were contacted only for the purpose of the study. All identity information was anonymised during analysis and reporting.

The interviews were conducted via telephone to ensure accessibility, as many participants lacked stable Internet or suitable devices for online interviews. Telephone interviews were the most inclusive method, helping to reduce digital barriers. According to Drabble et al. (2016), such methods are effective for gathering reflective responses from diverse and dispersed populations. Although telephonic interviews can be affected by environmental distractions (Farooq 2017), participants were contacted in advance to arrange a quiet time for the calls. Interviews were recorded with participants’ consent.

The first section of the interview captured demographic details and participants’ experience with teaching, caring for or managing LSB in special schools. The interviews were recorded using a cell phone, and field notes were taken (Cohen 2007). Data collected from section one of the interviews elicited the participants’ demographic details. The interviews explored recommendations to improve the care and management of LSB at the schools. The interviews were conducted until data saturation had been reached (data sufficiency) (Clarke & Braun 2019). Data saturation occurred when no new data emerged from the participants’ responses and data were no longer contributing to the development of new categories.

### Data analysis

The six phases of reflective thematic analysis, as described by Braun and Clarke (2006) were followed. *Familiarisation*: As the first step, the researchers familiarise themselves with the data, a process that included the process of reading and re-reading all transcripts to become familiar with the data. The audio recordings were manually transcribed verbatim to ensure accuracy and preserve participants’ original meanings. Transcripts were anonymised by removing identifiable information to maintain confidentiality. All data collected from the open-ended interviews, in-depth interviews and field notes were gathered and entered into a Microsoft Excel spreadsheet for analysis. *Coding*: The second step involved the generation of codes. The different codes on each of the interview transcripts were discussed by the researcher and two independent reviewers. The codes were organised to explore the relationship to categories and discover main themes and sub-themes. Two reviewers independently conducted initial coding, identifying preliminary themes. Discrepancies were resolved through discussion and re-examination of the data. A third reviewer was consulted to ensure consistency and alignment with the research questions, leading to a finalised coding scheme. *Generating initial codes*: Themes were constructed and organised by the first author. At the end of this step, themes were constructed based on the research objectives. A theme was defined as something that has a certain level of pattern or meaning concerning the research objective (Braun & Clarke 2006). *Review*: Potential themes were reviewed by the authors to reach an agreement on the codes and themes, ensure their alignment with the data and agree on their inclusion in the final report. Data credibility was assured by the development of a comprehensive data management system, which included a list of quotes, codes and themes. Data were reviewed and discussed to ensure meaning. The authors discussed the adequacy of the codes and themes within the context of the ICF until an agreement was reached. The data associated with each theme were read to check that the data supported the themes. *Definition and naming*: Feedback received from participants was combined according to the components of the ICF framework. The ICF framework was used to arrange codes from the data on the challenges of managing LSB and the recommendations to improve the care of LSB (Table 2 and Table 3). In inductive analysis, initial codes and themes were developed directly from the raw data using Braun and Clarke’s thematic analysis method. In the deductive analysis phase, the authors used the codes and themes to organise the data into the ICF categories to maintain alignment with the research objectives (WHO 2001). The ICF provided a concept structure to group and interpret the final themes. *Reporting*: The final report presents the synthesis of the qualitative findings.

**TABLE 2:** Challenges of managing learners with spina bifida.

ICF domain	Theme	Quotes
Body functions and structures	Infection related to spina bifida	‘The learners struggle with bladder and bowel, some of them struggle because of diarrhoea, especially after eating food with soup or beans.’ (Educator; Province A) ‘One of the biggest challenges is developing pressure sores and bed sores which are very frequent and very serious for these learners.’ (Nurse; Province B) ‘Learners have no skin sensation if the housemother is not around, they may burn from hot water, they use nappies and they have sores because they always sit on the wheelchair for too long.’ (Educator; Province A) Some of the learners do not feel anything when they are wet, and the moisture from bladder and bowel incidents causes them to have inflammation of the skin when in contact with urine. The learners also develop wounds because of taking too long to change pampers and sitting in one position for a long time, and these wounds take too long to heal. (Nurse; Province B)
Educational activity	Poor academic performance	‘Learners with spina bifida have short attention spans and they easily forget what they were taught in class. You have to repeat lessons most of the time for learners to understand and you find that you are always behind with your lessons.’ (Educator; Province B) ‘Some of the learners take time to reach where they are going because of poor mobility, they struggle to balance while walking.’ (Educator; Province B)
Participation in daily activities	Lack of independence	‘Some of the learners do not want to change [*their own*] diapers. They do not want to do anything by themselves because they are not used to doing things on their own. I think this is not good for their health.’ (Housemother; Province A) There are some learners who can be able to use their hands, but they do not want to bath themselves or learn to comb their hair; they always want to be assisted by the housemothers. (Housemother; Province A)
Environmental factors	Lack of resources	‘We do not have enough house mothers that can take care of learners. One house mother has to take care of more than 20 learners who all need help from one house mother.’ (Educator; Province A) ‘Shortage of equipment to be used by physiotherapists at the physio centre is a challenge because it affects the support to be rendered to learners by the physio.’ (Housemother; Province A) ‘We do not have enough devices to assist learners and some of the devices were expensive.’ (Educator; Province B) ‘Lack of occupational therapists, physiotherapists, speech therapists, social workers, and psychologist to support the learner is affecting the delivery of services to learners, especially because learners with spina bifida need more support from all this team.’ (Nurse; Province A) A learner who cannot be able to push themselves need an automatic wheelchair and is very expensive. (Educator; Province B)
Personal factors	Lack of knowledge	‘The most overlooked issue is the lack of knowledge; we might have the resources, but you don’t even know how these resources can be beneficial for learners with spina bifida. The problem is that we don’t know what to do.’ (Educator; Province B) ‘I studied to become a teacher, but when I was employed in a special school, I was not trained on how to teach these learners, is very challenging because you just teach.’ (Educator; Province B) ‘There are no house mothers who are trained to manage learners with spina bifida. We are using our own experience of having been working with learners.’ (Housemother; Province A) ‘I do not have a lot of information about spina bifida because I have not received training, if I can get training about the disability, I think it will make a good impact on learners because I will be able to prevent some of the preventative infection.’ (Educator; Province A) ‘We do not know how we can assist learners who are experiencing academic challenges because we were not trained on how to assist LSB. I studied to become a teacher, but when I was employed in a special school. I was not trained on how to teach these learners, is very challenging because you just teach.’ (Educator; Province A)
	Parental indifference and neglect	‘Parents do not bring learners on time when the school reopens, a learner can come back three weeks after the school has opened and they have lost a lot of lessons.’ (Educator; Province A) ‘Learners are coming back to school looking very thin and pale like they were not eating enough food.’ (Housemother; Province A) ‘There were delays in changing nappies and poor self-care when learners with spina bifida were at home. You will see the signs of neglect when they come back to school with wounds and pressure sores.’ (Purse; Province B) ‘For some of the learners, parents do not want to buy diapers, and this becomes a challenge for house mothers caring for the learners because a learner cannot stay without wearing a diaper.’ (Nurse; Province A) ‘Spina bifida learners receive disability grant and some parents abuse the learners’ grant, and they do not pay school fees.’ (Social worker; Province A)

ICF, International Classification of Functioning; LSB, learners with spina bifida.

**TABLE 3:** Recommendations to improve the care of learners with spina bifida.

ICF domain	Theme	Recommendations and quotes
Body functions and structures	Provision of infection control guidelines	Schools should implement a bladder and bowel management programme. ‘We should have bladder and bowel management during class so that the learners’ bodies can get used to relieving at a given time.’ (Occupational therapist; Province B) Daily checks for pressure sores and injuries should be conducted. ‘House mothers have to check learners every day to see if they are not developing sores.’ (Physiotherapist; Province A)
Activity in relation to performing school tasks	Train educators to support LSB in the classroom	Educators should receive training to support LSB academically. ‘Educators need training so that they can be able to support learners who are experiencing academic challenges in the classroom.’ (Occupational therapist; Province B) Educators should be trained to use strategies that are available when teaching learners with spina bifida. This training is important because it will help educators to understand how learners are taught. (Speech therapist; Province B)
Participation in daily activities	Introducing self-care and independence	Introduce age-appropriate self-care training for LSB to improve independence. ‘Learners have to be trained to care for themselves, they should learn to change diapers, and clothes, and to always stay clean.’ (Housemother; Province A) Educate parents through support groups. ‘Support groups must be offered to parents to educate them about infection control.’ (Nurse; Province B)
Environmental factors	Employment of adequate staff	Employ professional nurses, therapists and social workers with knowledge of spina bifida. ‘The schools need to employ professional nurses, social workers, occupational therapists, speech and physiotherapists who are trained to work with learners.’ (Educator; Province A) Increase the number of housemothers to reduce workload. ‘Schools should employ enough housemothers to care for learners.’ (Housemother; Province A) ‘We need to be capacitated as professionals about spina bifida and infection control, so we know how to work with learners with spina bifida and how to educate parents to care for their learners. Staff should also be workshop. Schools must also have resources so that our work can be easy. It should be a priority to train nurses because they play a big role in the learners. Learners cannot learn when they are not well, and the nurses are there to look after the learner’s health.’ (Nurse; Province A)
	Quality monitoring of schools	Regular monitoring of schools accommodating LSB should be conducted. ‘Learners with spina bifida require a high level of support, schools accommodating learners should be checked regularly.’ (Nurse; Province B) Implement quality improvement monitoring through expert visits. ‘Schools should always be visited by experts in the field of spina bifida.’ (Social worker; Province A)
Personal factors	Provide adequate training to staff and parents about spina bifida and infection control	Train staff on spina bifida and infection control. ‘We need to be trained about spina bifida and infection control so we can be able to assist learners.’ (Nurse; Province A) Organise workshops for parents to improve their understanding and attitude towards LSB care. ‘Schools should provide training and workshops to parents about spina bifida and the importance of caring for learners.’ (Nurse; Province B)

ICF, International Classification of Functioning; LSB, learners with spina bifida.

### Trustworthy in the study

Transcripts were read and re-read to fully understand participant’s responses. Emerging themes and interpretations were discussed with two experienced reviewers who were not involved in data collection. Their feedback helped to refine themes. Immediately after the interviews, member checking was done by rephrasing and summarising what each participant said.

### Data management

Data were systematically collected and organised in a structured format, with clear identifiers for each data source (e.g. participant information, date of data collection and province). All raw data were initially stored in a secure to ensure proper backup and data loss. To ensure rigour, a process of double-checking was implemented throughout the data management process. Regular meetings were held with colleagues to discuss the coding process, resolve discrepancies and maintain consistency in the analysis. A random sample of coded data was reviewed by a reviewer for validation purposes. All data were stored following institutional or ethical guidelines for data retention. After the retention period, data will be securely disposed of by deletion, ensuring that no sensitive information is left accessible.

### Ethical considerations

Approval to conduct the study was obtained from the Biomedical Research Ethics Committee of the University of KwaZulu-Natal (BREC/00000854/2019).

**Permission:** Permission to conduct the study was secured from the Gauteng and Limpopo Provincial Departments of Education, with gatekeeper approval from school principals.

**Consent procedures:** Audio-recorded informed consent using a cell phone was obtained from all participants who were informed of the study’s purpose, procedures and their right to withdraw at any time through a cell phone. Consent was sought before audio recordings.

**Confidentiality measures:** Participant anonymity was maintained through the removal of identifying information. Individual interviews were identified by assigning a unique code, for example P1 (participant 1), occupation to distinguish participants, while also ensuring that confidentiality is maintained. Data were securely stored in password-protected digital files and locked physical storage, accessible only to the research team.

## Results

In response to questions on challenges in managing LSB, six themes were identified and organised using the ICF framework. These include infection related to spina bifida, poor academic performance, the lack of independence, limited resources, the lack of knowledge and parental indifference or neglect (see Table 2).

Table 2 highlights the challenges of managing LSB, categorised by main themes and illustrative quotes from participants. Table 2 organises the challenges related to *Personal Factors*, highlighting the lack of knowledge among staff and the impact of parental indifference and neglect on LSB.

In addition, the study collected data on how to improve the care of LSB, and this is presented in the same ICF format. The five themes relating to the recommendations include the development of infection control guidelines, training of educators to support LSB in the classroom, introducing self-care and independence, employment of adequate staff, quality monitoring of the school and providing adequate training to staff and parents about spina bifida and infection control (Table 3).

## Discussion

In this section, we discuss the challenges raised and recommendations from the staff in relation to each of the six components of the ICF.

### Body functions and structures about infection related to spina bifida

Staff reported that LSB face persistent challenges with bladder and bowel incontinence, which contribute to frequent infections, wounds and pressure sores, particularly on the heels and buttocks. These complications often lead to absenteeism for medical treatment. Because of impaired skin sensation, many LSB are unaware of wetness or injury, increasing the risk of skin inflammation and hospitalisation. These findings align with existing research that identifies incontinence and reduced sensation as key factors in health complications and decreased quality of life for LSB (Lyder & Ayell 2008; Ottolini et al. 2013; Saavedra, Maclellan & Gray 2018; Santos, Lopes & Koyle 2017).

To mitigate these risks, staff recommended the implementation of structured bladder and bowel management programmes in schools. Suggested measures include scheduled toileting routines and daily skin checks by house mothers to identify early signs of pressure sores or injury. The development and consistent application of infection control guidelines supported by a multidisciplinary team is critical to preventing complications and promoting the overall well-being of learners (Ambartsumyan & Rodriguez 2018; Copp et al. 2015).

### Poor academic performance

Educators noted that many LSB experience academic difficulties, particularly in reading, handwriting and sustaining attention, even with repeated instruction. These challenges reflect research linking spina bifida to cognitive impairments, executive dysfunction and hydrocephalus, all of which can hinder academic performance (Burmeister et al. 2005; Dennis et al. 2006; Winning et al. 2022).

To address these barriers, staff recommended targeted training for educators focused on inclusive education strategies, differentiated instruction and condition-specific teaching methods. Such professional development enhances educators’ ability to adapt curricula and respond effectively to diverse learning needs (Department of Basic Education 2014; Hull 2005; Savolainen 2009). Training that addresses the unique educational challenges faced by LSB can significantly improve teaching effectiveness and support better academic outcomes.

### Lack of independence and participation in daily activities

House mothers from several schools reported that some LSB showed limited independence, often unwilling or unable to manage basic tasks such as changing their own disposable diapers. Staff felt that greater parental involvement in promoting self-care was necessary. This reflects findings in the literature, which highlight delayed social development and dependence in LSB, often exacerbated by overprotective parenting (Barf et al. 2003; Johnson 1997; Kritikos & Holmbeck 2020; Verhoef et al. 2004).

To address this, staff recommended implementing structured self-care and independence programmes tailored to the learner’s developmental stage and abilities. Much like teaching a child to ride a bicycle, where initial support gradually gives way to independence, these programmes aim to build confidence and autonomy step by step. In addition, staff proposed forming parent support groups to offer guidance on encouraging independence at home and managing health-related care. This approach is supported by evidence showing that self-management training improves both health outcomes and quality of life for individuals with spina bifida (Dicianno & Wilson 2010; Logan et al. 2020).

### Lack of resources as an environmental factor

Staff, particularly in Limpopo province, reported a severe lack of resources impacting the care and support of LSB. Shortages included key professionals such as occupational therapists, physiotherapists, speech therapists, psychologists and social workers as well as an insufficient number of house mothers. These gaps lead to limited access to consistent care and placed considerable strain on existing staff. In addition, schools lacked basic equipment such as wheelchairs, walking aids, catheters, diapers and hygiene products, along with limited transport and communication resources for allied health staff. These deficits negatively affected learners’ mobility, hygiene, independence and overall well-being, reflecting broader rural–urban disparities in school resourcing and specialist availability (Department of Basic Education 2014; Sumbane et al. 2023).

To address these challenges, staff recommended implementing regular quality monitoring systems in special schools to ensure the consistent delivery of appropriate services. They also called for the recruitment of adequately trained professionals and support staff with expertise in spina bifida. These measures would support the goals of South Africa’s Inclusive Education policy, which advocates for equitable access to support and resources for all learners with disabilities (Department of Basic Education 2001).

### Personal factors and the lack of knowledge about spina bifida and infection control

Many staff members, especially house mothers in Limpopo province, reported limited knowledge about spina bifida and infection control practices. This lack of understanding affected their confidence and capacity to support LSB effectively. Without proper training, staff risk providing inadequate care, increasing the likelihood of preventable infections and health complications. These findings reflect broader concerns in the literature that caregivers often lack the training needed to support children with disabilities appropriately (Bannink, Idro & Van Hove 2016). This aligns with best practices in inclusive education and disability care, which emphasise capacity building among caregivers to improve learner outcomes (Department of Basic Education 2014).

### Parental indifference and neglect

Staff also reported that some parents were disengaged or neglectful in supporting their children. Behaviours included late returns to school after holidays, missed meetings, visible signs of poor care and refusal to provide necessary items such as diapers. These actions negatively impacted learners’ health, hygiene, emotional well-being and educational progress. This aligns with concerns in the literature about the misuse of care dependency grants and the consequences of neglect (Bannink et al. 2016; Trafford & Swartz 2023).

Staff suggested creating parent support groups and offering educational programmes focused on disability care, infection management and the importance of parental involvement. These interventions could improve home care practices and strengthen the partnership between schools and families. Raising awareness among parents is crucial for ensuring the well-being and development of LSB.

### Recommendations from this study

Based on the findings of this study, a number of integrated recommendations are proposed to improve the care, education and infection control management of LSB in special school settings. These recommendations span across policy, education and practical implementation levels, and implemented in a coordinated manner will create more inclusive, supportive and effective environments for LSB.

At the *policy level*, there is a need to develop and implement national guidelines specifically focused on the management of LSB in school settings. These guidelines should address both infection control and educational support, ensuring that consistent and evidence-based practices are applied across all schools. In addition, establishing quality monitoring systems is crucial. These systems should include regular inspections of facilities, teaching approaches and the availability of essential resources such as assistive devices and hygiene supplies. Furthermore, the government must allocate sufficient funding to recruit and retain qualified staff, including healthcare professionals, therapists and housemothers, to ensure that LSB receive comprehensive support.

At the *educational level*, it is essential to integrate training on teaching of LSB and other disabilities and inclusive education practices into pre-service and in-service teacher training programme. Educators need to be equipped with the knowledge and practical skills to support the diverse learning and care needs of LSB. Professional development initiatives, such as workshops and seminars, should also be offered to healthcare staff and parents, focusing on infection prevention, promoting independence and understanding the needs of LSB. In addition, school curricula should be adapted to accommodate the specific cognitive, physical and social challenges faced by LSB, ensuring they have equitable opportunities to succeed academically.

At the *practice level*, schools should establish multidisciplinary teams comprising nurses, occupational therapists, physiotherapists, social workers and educators. These teams can work collaboratively to provide holistic care and support for LSB, addressing both health and learning needs. Infrastructure within schools must also be reviewed and improved to ensure accessibility, proper hygiene and the availability of mobility aids and other assistive technologies. Collaboration between schools, healthcare providers and families should be strengthened to ensure coordinated care. In addition, awareness-raising campaigns within school communities are recommended to reduce stigma and promote understanding of spina bifida among learners and staff.

### Limitations of the study

The following limitations were noted in this study. The interviews were conducted telephonically. One of the disadvantages of a telephonic interview is that the interviewer does not see the interviewee, so body language cannot be recorded in field notes as additional information (Opdenakar 2006). In this study, the large volume of qualitative data collected posed challenges during data analysis, particularly in ensuring consistency in the interpretation of responses. To address this, the deductive framework of the ICF provided a framework for analysis and reporting.

### Implications for future research

Future research should focus on assessing the long-term effects of implemented policies and practices on the health and educational outcomes of LSB. Studies should also explore effective strategies to enhance parental involvement and adherence to recommended care practices, as their engagement is crucial for consistent support. In addition, research should identify cost-effective interventions tailored for resource-limited settings to improve the care and education of LSB, ensuring equitable access to quality services despite financial constraints.

## Conclusion

This study highlights systemic gaps in infection control readiness for LSB in schools, particularly in low- and middle-income countries (LMICs). Poor staff knowledge, inadequate resources and limited parental involvement exacerbate infection risks, impacting LSB’s health, academic performance and independence. Addressing these deficiencies through targeted training, resource allocation and policy reform can create a safer, more inclusive environment for LSB. These findings offer a foundation for future research and interventions to improve the well-being and educational outcomes of LSB.
